# Investigating the Direct Impact of a Gamified Versus Nongamified Well-Being Intervention: An Exploratory Experiment

**DOI:** 10.2196/jmir.9923

**Published:** 2018-07-26

**Authors:** Saskia Marion Kelders, Marion Sommers-Spijkerman, Jochem Goldberg

**Affiliations:** ^1^ Center for eHealth and Wellbeing Research Department of Psychology, Health and Technology University of Twente Enschede Netherlands; ^2^ Optentia Research Focus Area North-West University Vanderbijlpark South Africa

**Keywords:** gamification, well-being, engagement, electronic mental health, mental health

## Abstract

**Background:**

Gamification is a promising strategy to increase the effectiveness of Web-based mental health interventions by enhancing engagement. However, because most studies focus on the longer term effects of gamification (eg, effectiveness or adherence at the end of the intervention period), there is limited insight into how gamification may enhance engagement. Research implies that gamification has a direct impact at the time of use of the intervention, which changes the experience of the users, and thereby motivates users. However, it is unclear what this direct impact of gamification might be and how it can be measured.

**Objective:**

The objective of this study was to explore the direct impact of gamification on behavioral, cognitive, and affective engagement in the context of a Web-based mental health intervention and to explore whether and how the different components of engagement are related.

**Methods:**

A pilot (n=19) and a real-life (n=75) randomized between-groups experiment was carried out, where participants used a gamified or nongamified version of the same Web-based well-being intervention for a single session. Participants (68%, 64/94 female, mean age 23 years) were asked to use the intervention in one session for research purposes. Gamification elements included a map as visualization of the different lessons, a virtual guide, and badges. Later, behavioral, cognitive, and affective engagement were measured.

**Results:**

The pilot experiment showed no differences between the gamified and nongamified intervention. However, in the real-life experiment, participants in the gamified intervention scored higher on cognitive engagement, that is, involvement (*P*=.02) and some elements of affective engagement, that is, flow as a combination of cognitive and affective engagement (*P*=.049), and the emotions ”interest” (*P*=.03) and “inspiration” (*P*=.009). Furthermore, the effect of gamification on cognitive engagement was mediated by the influence of gamification on specific positive emotions.

**Conclusions:**

The gamified intervention seemed to be able to increase cognitive engagement and the combination of cognitive and affective engagement but not behavioral and affective engagement alone. However, positive emotions seem to play an important role in mediating the effect of gamification on engagement. In conclusion, we cannot say that gamification ”works” but that the design of an intervention, in this case, gamification, can have an impact on how participants experience the intervention.

## Introduction

### Background

Web-based interventions, in which people can improve their health from home, with or without the help of a health care professional, are increasingly used in many health care areas [1-3]. Advantages of Web-based interventions compared with face-to-face interventions are, among others, that Web-based interventions can reduce the costs of providing interventions, increase access to care for a large group of people, and are often perceived as more convenient by the users, given their flexibility and anonymity [4,5]. These interventions have been shown to be effective, for example, decreasing depressive symptoms, increasing well-being, and stimulating people to become more active [2,6-10]. However, not all Web-based interventions show beneficial effects and especially the effect sizes of interventions implemented outside the clinical setting with limited or no counselor involvement can be quite small [7,8,10].

The limited effectiveness of these interventions may be partly attributed to large nonadherence rates [11,12]. Many people who start using a Web-based intervention do not finish it or do not use the intervention in the prescribed way, which diminishes its effectiveness [13]. More recently, it has been posited that adherence (ie, using the intervention as intended by the developers) alone may not be enough for an intervention to be effective but that it is also necessary for participants to feel involved with an intervention or to be able to identify with the intervention [14,15]. Together, these factors may be called engagement, and it seems that a certain level of engagement is required for an intervention to be effective [16]. Research has shown that technology offers ample opportunities for enhancing engagement [12,17]. Gamification is one of these technological opportunities and is increasingly recommended and used to make interventions more engaging [18-21].

Gamification has been defined as “using game design elements in nongame contexts” [22]. These game design elements can be very specific, for example, the inclusion of “badges” or “levels” in the interface. They can also be broad, for example, including a storyline to make the goals clear and stimulate enduring play. The nongame part of the definition refers to gamification not being a full-fledged game, as opposed to serious games. The main goal of gamification is to increase participants’ engagement with the intervention. Multiple studies have shown the potential of gamification to increase adherence to and effectiveness of health interventions, for example, a mobile intervention for mental health [23] and a Web-based intervention for physical activity [24]. Nonetheless, many of these studies have methodological limitations and merely focus on adherence or effectiveness, thereby shedding limited light on whether and how gamification affected these variables [18,25]. Authors have indicated the need for more comparative studies (gamified vs nongamified versions of the same intervention) to isolate the effects of gamification [25].

A number of studies have shown that gamification, if used correctly, can increase intrinsic motivation for a certain behavior, for example, by satisfying certain psychological needs [26,27]. However, although motivation can be seen as necessary, it is not sufficient to achieve engagement [28]. Engagement may be seen as a multidimensional construct, consisting of behavioral, cognitive, and affective components [28,29]. In the case of Web-based health interventions, the behavioral component may refer to the usage of or adherence to the intervention [16]. It seems likely that gamification has an impact on the time participants spend in each session with the intervention, the number of exercises they perform, or how elaborately they complete each of the exercises. The cognitive and affective components are less well understood in the context of Web-based health interventions. According to a recent review, these components are often taken together as a subjective experience characterized by attention, interest, and affect [16]. Despite limited knowledge on what may constitute cognitive engagement in Web-based health interventions, from the student engagement context, in which cognitive engagement has been described as “students’ psychological involvement in learning” [30], the concept of involvement becomes apparent. Involvement relates to the importance of a product (eg, an intervention) to the individual [31] and is an important predictor of the effectiveness of Web-based mental health interventions [15]. Enjoyment is an important motivator for people to use games and might also be integral to the affective engagement with gamified interventions [19]. Enjoyment is also closely related to the concept of intrinsic motivation as a part of the self-determination theory [32]. This theory has been used to explain the appeal of games [33]. Positive emotions may also be a part of affective engagement. Because positive emotions lead to a broadening of one’s attention [34], these emotions may be especially relevant to achieve in a Web-based health intervention, wherein users are taught new ways to deal with situations. Lastly, the concept of flow is a concept related to engagement. Flow is defined as “a mental state of operation, in which a person performing an activity is fully immersed in a feeling of energized focus, full involvement, and enjoyment in the process of the activity” and is often used as a way to explain gaming behaviors [35,36]. Because it incorporates both feelings of involvement and enjoyment, it may be a state wherein both cognitive and affective engagement come together.

According to Nicholson [20], gamification can only be beneficial if it provides a “positive and meaningful game-based experience” to its users, leading to a long-term engagement. This experience seems closely related to the cognitive and affective components of engagement. It implies that gamification directly impacts the experience of the user while he or she uses the intervention. This impact should already be made during the first use of an intervention. However, as most studies focus on the longer term effects of gamification (eg, effectiveness or adherence at the end of the intervention period), little is known about the direct impact of gamification on engagement or how systems should be designed to foster this direct impact.

### Objective

The goal of this study was to explore the direct impact of gamification on behavioral, cognitive, and affective engagement in the context of a Web-based mental health intervention and to explore whether and how the different components of engagement are related. To achieve this goal, an exploratory randomized experiment was carried out where participants used a gamified or nongamified version of the same Web-based positive psychology intervention in a single session. In terms of content, both versions of the intervention were identical.

The intervention used in this experiment seeks to improve well-being. Well-being is important to achieve and maintain a healthy life and prevent mental illnesses and generally serves as a basis for resilience [37-40]. Research has shown that well-being can be improved through training and the specific intervention used in this study has also been proven effective in improving well-being. Following the positive effects of the intervention as email guided bibliotherapy [41], a Web-based version was created [42]. Although this Web-based intervention offered the opportunity to enhance the scalability of the intervention against limited costs, specific attention should be paid to engaging participants. Hence, this intervention was deemed as an ideal candidate for gamification.

## Methods

### Design

A between-groups experimental design was used. For the study, 2 versions of the same intervention were created (ie, a gamified version and a nongamified version). Although both versions contained the same information and exercises (ie, same texts), the information and exercises were presented in a different manner. A pilot experiment was performed in a lab setting to check the procedure and the versions of the interventions before the actual experiment was carried out in a more real-life setting. The pilot study focused on investigating the experimental procedures, not the intervention, which was pilot-tested before [42]. Our aim was to test whether participants could use the intervention without any guidance and foreknowledge in a meaningful way in one session. Therefore, the experimenters were nearby while participants used the intervention, after which they were briefly asked about their experiences. However, this formal setting seemed to influence not only the type of participants (ie, the pilot attracted mainly students who were already interested in positive psychology) but also the way they used the intervention (ie, the pilot participants used the intervention in a very focused setting without any distractions). Because this is not how the intervention will be used in real life, we decided on a different setting for the real-life experiment.

### Recruitment and Participants

The study population consisted of people aged 18 years or older. Exclusion criteria were insufficient proficiency in the Dutch language (reading and writing) and the inability or unwillingness to provide informed consent. Because the University of Twente has a large proportion of German students who have learned Dutch for their studies, people of Dutch and German nationalities were able to participate as long as they had sufficient proficiency in the Dutch language. Recruitment for the pilot experiment was done through the University of Twente research participants system. Bachelor Psychology students need to earn “participant points” by participating in research studies. Overall, 19 psychology students from the University of Twente participated in the pilot experiment, of which 11 were randomized to the gamified intervention and 8 were randomized to the nongamified intervention (Table 1). Analyses showed a significant difference in nationality between the conditions with more Dutch than German participants receiving the gamified intervention (*χ*^2^_1_=8.7; *P*=.003).

For the real-life experiment, participants were recruited through the University of Twente research participation system and through convenience sampling by undergraduate psychology students who assisted in conducting the experiment as part of a Bachelor research project. Overall, 76 participants were included in the study and randomly allocated to receive either the gamified intervention (n=39) or the nongamified intervention (n=37).

One respondent in the nongamified condition had to be excluded from the analysis owing to an issue with the account the participant used for the experiment (the account had been used before so the participant was not able to complete the experimental procedure). Table 1 provides an overview of the participants included in the analyses. There were no significant differences in the demographic characteristics between the conditions.

Additional analyses showed that participants in the real-life experiment were older (22.8 vs 19.6; *F*_1,92_=10.053; *P*=.002) and more often German (77%, 58/75 vs 37%, 7/19; *χ*^2^_1_=11.7; *P*=.001) compared with those included in the pilot experiment. There was no significant difference in gender between the pilot and real-life experiments (*χ*^2^_1_=1.3; *P*=.26).

### Power Analysis

G*Power 3.1.9 (Heinrich Heine Universität, Düsseldorf, Germany) was used to calculate the required sample size for detecting a medium effect (Cohen *d*=0.5) in an independent samples *t* test (two-tailed). With 80% power at an alpha level of .05, a total sample size of 128 participants (64 per group) was needed to test the hypotheses. Unfortunately, recruitment turned out to be difficult and we did not manage to reach our intended number of participants.

### Intervention

The intervention used for this experiment was called “This is your life,” a Web-based positive psychology intervention which aims to improve well-being in the general population [43] and has been proven effective as a self-help book with email counseling [41]. The Web-based gamified intervention was developed using a human-centered design [34]. Potential users that participated in the codesign process indicated the potential value of gamification and cooperated in designing the specific gamification features. Following their recommendations, we decided that the main storyline would be a user on a journey toward a flourishing life, guided by a professor. The intervention consisted of an introduction and 8 lessons that could be completed in 12 weeks. Each lesson consisted of psychoeducation and approximately 5 exercises that could be completed multiple times. In each lesson, there were approximately 2 key challenges; these were the exercises that needed to be completed to be able to continue to the next lesson.

**Table 1:** Participants’ demographic characteristics.

Demographics		Gamified pilot (n=11) and real-life (n=39)	Nongamified pilot (n=8) and real-life (n=36)	Total pilot (n=19) and real-life (N=75)	Statistics		
					*F* value	χ^2^_1_	*P* value
**Age (years), mean (SD)**							
	Pilot	19.6 (1,7)	20.0 (1.3)	19.7 (1.5)	0.399^a^	—	.54
	Real-life	23.4 (5.6)	22.2 (1.5)	22.8 (4.2)	1.610^b^	—	.21
**Sex (female), n (%)**							
	Pilot	8 (73)	7 (88)	15 (79)	—	0.6	.44
	Real-life	28 (72)	21 (58)	49 (65)	—	1.5	.22
**Nationality (Dutch), n (%)**							
	Pilot	10 (91)	2 (25)	12 (63)	—	8.7	.003
	Real-life	10 (26)	7 (19)	17 (23)	—	0.4	.52

^a^*F*_1,17_

^b^*F*_1,73_

The intervention was completely self-guided; there was no guidance or feedback from a human counselor. However, the intervention itself did provide tailored feedback when a user finished a lesson and provided general feedback about how to best perform exercises at various points during each lesson. For the experiment, participants were asked to complete the introduction and 2 exercises from the first lesson in one session. These 2 exercises were “Three good things” (relive and write about 3 good things that happened today) and “Write about positive experiences” (relive and write about a beautiful memory from one’s own life).

#### Gamified and Nongamified Version

As stated earlier, both versions of the intervention contain the same information and exercises. Differences were only in lay-out and in wording of feedback, as indicated in the next section.

##### Lay-Out of the Intervention Overview

**Figure 1:**
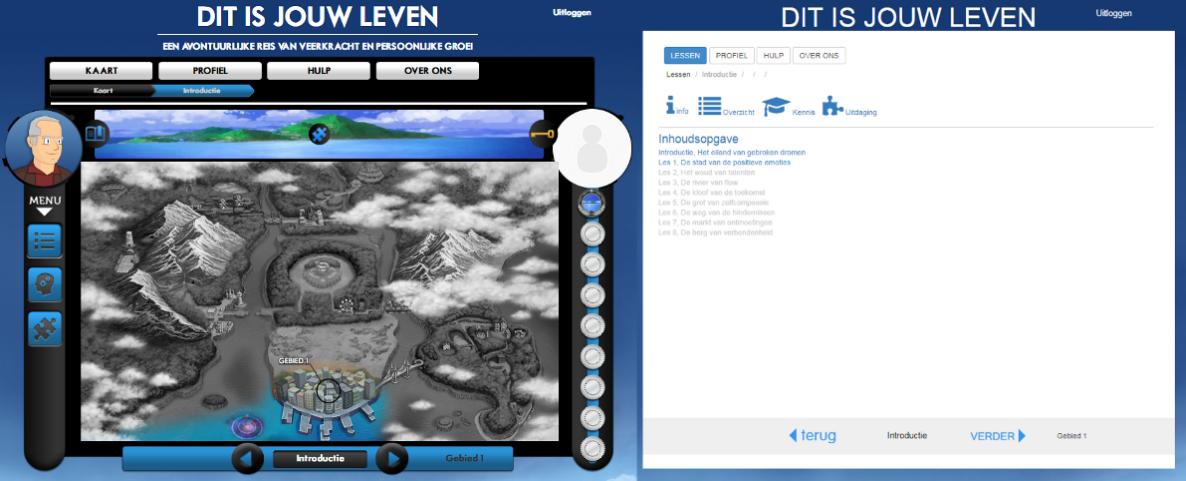
Overview of the intervention in the gamified version (left) and nongamified version (right). Source: University of Twente, Centre for eHealth and Wellbeing Research.

In the gamified version, the overview was visualized as a map, in which the participants travel to various destinations (the different lessons). In the nongamified version, a list of lessons was provided. In both versions, the lessons that could not yet be accessed were grayed out, as seen in Figure 1.

##### Lay-Out of the Lesson Screen

**Figure 2:**
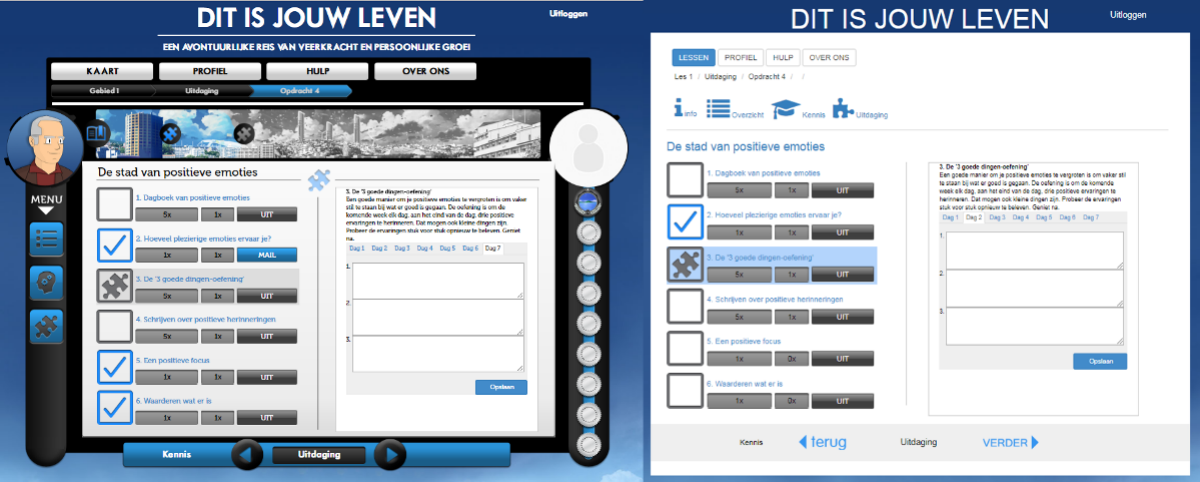
Lesson screen in the gamified version (left) and nongamified version (right). Source: University of Twente, Centre for eHealth and Wellbeing Research.

The basic features of the lesson screen were the same in both versions (list of exercises on the left and explanation and filling out opportunity on the right, as seen in Figure 2). The gamified version showed an additional progress bar, in which the activities of the lesson were visualized; each time a mandatory activity was completed, a part of the progress bar was colored in. After finishing all the mandatory activities, participants in the gamified condition were granted a key with which they could enter the next destination. Participants in the nongamified condition were provided with a link to start the next lesson after completing the mandatory activities.

##### Professor and Participant Avatar

In the gamified version of the intervention, participants were guided through the intervention by an avatar of “Professor Happiness,” as seen in Figures 1 and 2. Instructions and feedback appeared as a pop-up coming from the avatar. In the nongamified version, the same instructions and feedback were given through a pop-up of the info-button. The wording used in both versions was slightly adapted to appear to come from the “Professor” (eg, using “I”) or from “info” (eg, using the passive form). In the gamified version, there was also room for a participant avatar (or photo), but this feature was not used in the experiments.

##### Badges

Participants in the gamified version earned a badge after completing the introduction and each of the lessons. These badges were shown on the right side of the screen, as seen in Figures 1 and 2. When “mousing over” these badges, a quote matching the badge’s lesson was shown. Because participants in the experiments only needed to complete the introduction and some exercises in the first lesson, participants typically only saw the badge which was awarded following the completion of the introduction. The quote for this first badge was “It is good to have an end to journey toward; but it is the journey that matters, in the end–Ernest Hemingway.”

#### Randomization and Blinding

In both the pilot and real-life experiments, participants were randomly assigned to the gamified or nongamified intervention. A random number list was created (using random.org) and participants were allocated according to this list, in the order in which they registered for the study. Randomization was double blind; the experimenters did not know to which condition the participants were assigned and the participants did not know that different versions of the intervention existed.

### Procedure

For the pilot experiment, participants were asked to come into the lab and were seated in a cubicle, where a personal computer was set up with both the log-on page to the intervention and the questionnaire opened in a separate tab of the Internet Explorer browser. Participants gave informed consent before receiving the log-in details and instructions. The experimenter was not in the room when the participants performed the experiment but was available in case the participants experienced any (technical) issues.

In the real-life experiment, participants were made to meet face-to-face (in their home or at the University) or online to participate in the experiment. Participants gave informed consent before receiving the log-in details and the explanation of the procedure. Participants could complete the experiment at their leisure but were instructed to follow the procedure given to them.

In both the experiments, participants were asked to log into the intervention using the account details provided and to complete four tasks within the Web-based intervention in one session, regardless of condition. The four tasks were as follows: complete the tutorial, read the information of the first module, complete the exercise “Three good things” once, and complete the exercise “Write about positive experiences” once. These were the tasks we expected new users to complete during their first session. After completing these tasks, participants were free to further explore the intervention or to end the session. Completing the aforementioned tasks typically took approximately 30 minutes. After ending the session, participants were asked to fill out a Web-based questionnaire. Log data were used to assure that all participants conformed to the procedure (using the intervention for only one session and filling out the questionnaire after ending the session). Prior to the study, ethical approval was obtained from the Faculty of Behavioral Sciences Ethics Committee at the University of Twente.

### Measures

Behavioral engagement was assessed by means of usage measures (ie, time spent on the intervention, the number of exercises completed, and the number of words used) gathered through system logs (log data). For the calculation of the time spent on the intervention, “log-in” time was considered as the start time and the time when the last action was performed was considered as the end time. For this last action, it was decided that the “log-out” time would not be considered because many participants never logged out and many logged out much later (eg, 20 minutes) after performing the last action, indicating that they were not actively using the system at that time anymore, for example, completing the Web-based questionnaire. For one participant, the number of words used could not be retrieved because of a technical error.

After completing the tasks within the intervention, participants filled out a Web-based questionnaire measuring cognitive and affective engagement. For cognitive engagement, involvement was measured with the short version of the Personal Involvement Inventory (10 items, mean score 1-7, higher score means more involvement [31]). For affective engagement, positive emotions were measured with the corresponding items of the Positive And Negative Affect Schedule (PANAS, 10 items, total score 5-50, higher score means more positive emotions [44]). Enjoyment was measured with the enjoyment subscale of the Intrinsic Motivation Inventory (IMI, 7 items, mean score 1-7, higher mean score means more enjoyment [45]). Lastly, to measure both cognitive and affective engagement, flow was measured with the newly established Flow State Questionnaire of the Positive Psychology Lab (PPL-FSQ; 20 items, mean score 1-5, higher mean score means more flow [46]).

Furthermore, overall satisfaction with the intervention was assessed by asking the participants to grade the intervention from 1 to 10; higher scores indicate greater satisfaction with the intervention. Finally, in the real-life experiment, but not in the pilot test, usability was measured using the System Usability Scale (SUS, 10 items, total score 0-100, higher score means higher usability [47]). For all measures, Cronbach alpha values varied between.83 and.94 based on the data of both studies.

### Analyses

Statistical analyses were performed using SPSS 23 (IBM, USA). All tests were two-tailed and the value for alpha was .05. Differences between conditions with regard to the outcome variables were investigated using one-way analysis of variance. Effect sizes are presented as Cohen *d*. Exploration of differences between conditions on single items of questionnaires (instead of on mean or sum-scores) were done using Mann Whitney *U* tests because of the ordinal level of this data from Likert-scale questions.

Within the pilot experiment, the data showed signs of not being normally distributed. Shapiro-Wilk tests were significant for the measurement of enjoyment, flow, and the number of words and exercises. Therefore, bootstrapped 95% CI of the mean differences were calculated for all outcome measures in the pilot experiment.

To explore whether and how the components of engagement were related, exploratory simple mediation analyses were conducted. More specifically, we investigated whether the influences of gamification on involvement and flow, which seems to be an effect that needed some conscious effort, was mediated by positive emotions, which seemed to be a more direct and effortless effect. A subscale of PANAS was created with items that are expected to be influenced by the gamified design (ie, interested, enthusiastic, inspired, and attentive). Mediation analyses were performed using the PROCESS macro for SPSS [48]. For each outcome (ie, involvement and flow), a separate mediation analysis was conducted. Condition (gamified or nongamified) was entered as the predictor variable, the PANAS subscale as the mediator, and either involvement or flow as the outcome variable. To test whether the indirect effect is statistically different from zero, 10,000 bootstrap CI were generated. When the corresponding bias-corrected 95% bootstrap CI did not include zero, the indirect effect was considered significant.

## Results

The results of the pilot experiment conducted in the laboratory are presented in Table 2. Overall, the participants highly valued the intervention with an average grade of 7.8 and spent approximately 30 minutes using the intervention.

Table 3 presents the results of the real-life experiment. Significant differences were found for involvement and flow, whereby the gamified condition scored higher with approximately a medium effect size (*F*_1,73_=5.919; *P*=.02 and *F*_1,73_=4.626; *P*=.04; respectively). The total score of positive emotions did not show significant differences. Due to the various positive emotions that are measured with PANAS, we performed exploratory analyses to investigate whether there were any significant differences in distinct emotions (ie, single items of the PANAS questionnaire). With regard to the emotions “interest” and “inspiration,” it was found that the gamified condition scored significantly higher than the nongamified condition (Z=−2.239; *P*=.03 and Z=−2.454; *P*=.01; respectively). There were no significant differences in the other distinct emotions. For enjoyment, usability, intervention satisfaction, and different usage measures, no significant differences were observed.

**Figure 3:**
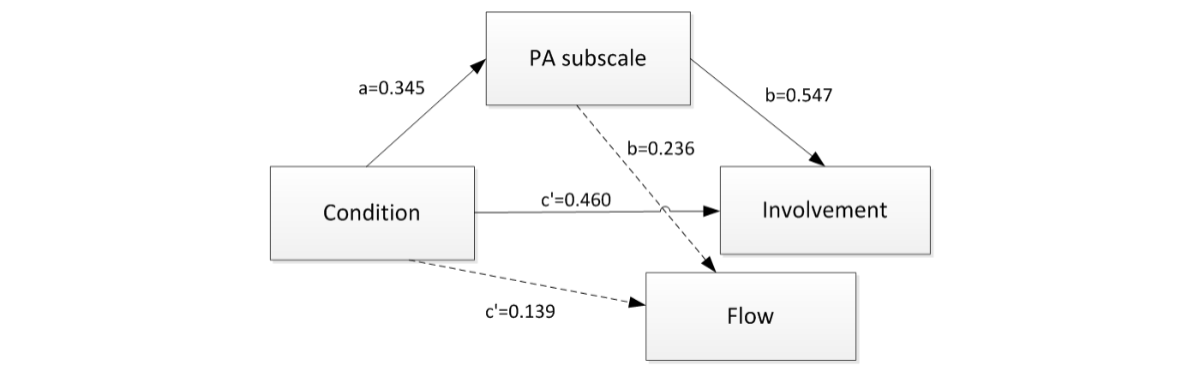
Mediation models. PA: positive affect.

Simple mediation analyses showed that the condition indirectly influenced both involvement and flow through its effect on certain positive emotions. As seen in Figure 3 and Table 4, participants in the gamified condition scored higher on positive emotions (a=0.345), and participants who experienced more of the positive emotions interest, enthusiasm, inspiration and attentiveness, scored higher on both involvement (b=0.547) and flow (b=0.236). For both outcomes, a bias-corrected bootstrap 95% CI for the indirect effect (involvement, ab=0.188; flow, ab=0.081) based on 10,000 bootstrap samples was completely above zero (involvement, 0.017 to 0.539, flow, 0.011 to 0.204). There was no evidence that the condition influenced involvement or flow independent of the effect on the positive emotions (involvement, c'=0.460; flow, c'=0.139).

**Table 2:** Pilot experiment outcome variables.

Outcome	Gamified (n=11), mean (SD)	Nongamified (n=8), mean (SD)	Total (N=19), mean (SD)	Effect size (*d*)	*F*_1,17_	*P* value	Bootstrapped 95% CI of mean difference
Positive affect	36.00 (6.01)	34.25 (2.71)	35.26 (4.87)	0.38	0.583	.46	−5.86 to 3.15
PII^a^	4.78 (1.16)	5.38 (1.10)	5.03 (1.14)	−0.53	1.271	.28	-0.53 to 1.60
IMI-E^b^	4.81 (1.23)	5.66 (1.17)	5.17 (1.25)	−0.71	2.341	.14	−0.36 to 1.91
PPL-FSQ^c^	3.61 (0.36)	3.77 (0.45)	3.68 (0.39)	−0.39	0.748	.40	−0.18 to 0.57
Satisfaction	7.55 (1.21)	8.25 (1.58)	7.84 (1.34)	−0.50	1.213	.29	−0.64 to 1.94
Number of exercises	7.55 (7.97)	3.63 (1.19)	5.89 (6.31)	0.69	1.877	.19	−8.92 to 0.21
Number of words	261.90 (271.21)^d^	205.00 (73.59)	236.61 (204.98)	0.29	0.329	.57	−244.10 to 74.79
Time	25.18 (11.94)	32.00 (7.45)	28.05 (10.62)	−0.69	2.018	.17	−2.38 to 15.97

^a^PII: Personal Involvement Inventory.

^b^IMI-E: Intrinsic Motivation Inventory, subscale Enjoyment.

^c^PPL-FSQ: Flow State Questionnaire of the Positive Psychology Lab.

^d^Based on n=10.

**Table 3:** Real-life experiment outcome variables.

Outcome	Gamified (n=39), mean (SD)	Nongamified (n=36), mean (SD)	Total (N=75), mean (SD)	Effect size (*d*)	*F*_1,73_	*P* value	
Positive affect	33.67 (5.47)	31.08 (7.19)	32.43 (6.44)	0.41	3.094	.08	
PII^a^	4.82 (1.06)	4.18 (1.25)	4.51 (1.19)	0.55	5.919	.02	
IMI-E^b^	4.71 (1.20)	4.17 (1.41)	4.45 (1.33)	0.41	3.156	.08	
PPL-FSQ^c^	3.54 (0.44)	3.32 (0.45)	3.44 (0.45)	0.49	4.626	.04	
SUS^d^	43.59 (14.89)	45.76 (21.49)	44.63 (18.26)	−0.12	0.263	.61	
Satisfaction	6.69 (1.70)	6.36 (1.90)	6.53 (1.80)	0.18	0.634	.43	
Number of exercises	4.41 (3.22)	4.36 (3.08)	4.39 (3.13)	0.02	0.005	.95	
Number of words	188.10 (112.90)	164.89 (101.87)	176.96 (107.66)	0.22	0.869	.35	
Time	26.95 (13.57)	29.06 (11.65)	27.96 (12.64)	−0.17	0.516	.48	

^a^PII: Personal Involvement Inventory.

^b^IMI-E: Intrinsic Motivation Inventory, subscale Enjoyment.

^c^PPL-FSQ: Flow State Questionnaire of the Positive Psychology Lab.

^d^SUS: System Usability Scale.

**Table 4:** Outcomes of the mediation models.

Variable		Coefficient	Standard error	*P* value
**Positive affect subscale^a^**				
	Condition (a)	0.345	0.161	.04
	Constant (i_1_)	3.271	0.1163	<.001
**Involvement^b^**				
	Condition (c')	0.460	0.261	.08
	Positive affect subscale (b)	0.547	0.184	.004
	Constant (i_2_)	2.388	0.628	.003
**Flow^c^**				
	Condition	0.139	0.099	.16
	Positive affect subscale	0.236	0.070	.001
	Constant	2.549	0.237	<.001

^a^R^2^=.059; *F*_1,73_=4.566, *P*=.04.

^b^R^2^=.176; *F*_2,72_=7.680, *P*=.009.

^c^R^2^=.190; *F*_2,72_=8.424, *P*=.005.

## Discussion

### Principal Findings

To our knowledge, this is the first study that compared the direct impact of 2 mental health interventions that have the same content and only differ in the use of gamification. This exploratory study suggests that a gamified system, in a single session, can have a positive impact on cognitive engagement by increasing the involvement participants feel with the intervention. Participants also experience more flow when working with the gamified intervention. This points toward an increase in the combination of cognitive and affective engagement. However, the gamified intervention did not seem to increase behavioral or affective engagement as such. Furthermore, gamification did not have an (negative) influence on the usability of the system. Therefore, in this study, the gamified elements did not seem to add more complexity to the system, as observed in other studies [49].

We did not see any significant differences between the conditions in the pilot experiment. Most likely, this was a power issue because there were very few participants in this experiment. However, the goal of the pilot experiment was to test the procedure of the experiment and based on the experiences of participants, changes were made in the actual study. The pilot study served its purpose.

Looking closer at the results of the real-life experiment, gamification did not seem to increase affective engagement compared to the nongamified intervention. This might be because a gamified intervention is not a game (which might be played for enjoyment and a positive experience) but only leverages some of the game design aspects. The results on the distinct positive emotions corroborate this finding: only interest and inspiration were significantly improved by gamification. A possible explanation for this finding is that these emotions relate more to the meaningfulness of the experience than to its valance. Moreover, we found that the impact of gamification on cognitive engagement was mediated by positive emotions that we expected to be influenced by the gamified design. This indicates that specific positive emotions (ie, interested, enthusiastic, inspired, and attentive) do play a role. The gamified design that was used in this study seemed to leverage these positive emotions to increase cognitive engagement with the intervention. However, because we assessed all measurements at the same time, we cannot be certain that gamification first increased these emotions, which in turn affected cognitive engagement.

Gamification did not seem to increase behavioral engagement. However, this may not be surprising because more is not always better [50]. Behavioral engagement was measured with usage data, as is common in research on Web-based interventions [16]. However, the amount of usage of a system does not always indicate the quality of usage. For instance, in this study, we asked participants to complete the activities that are likely to be sufficient for a first usage of the system. It might not be beneficial to do more exercises because you might not be able to put in the mental effort to do these extra exercises. Therefore, measuring behavioral engagement as purely the quantity of usage might not be ideal. However, because it is difficult to directly observe how participants use a Web-based intervention, especially in real life, it might not be feasible to measure the quality of behavioral engagement.

### Limitations

This study has some limitations. First, this study explored the direct impact of gamification on engagement. Although we feel that this is a necessary step to start understanding the impact of gamification, we acknowledge that our findings do not address the contribution of gamification to the effectiveness of and adherence to the intervention. Longer term studies are needed for this goal. Second, in this study, we have attempted to measure whether the gamified intervention increased cognitive and affective engagement because these aspects of the experience are posited as important for gamification to have a positive impact. However, in the literature, it is not yet defined what actually constitutes cognitive and affective engagement. Therefore, we have chosen measures that are related to these concepts and seem applicable to gamification, but there may be other measures that capture these concepts as well. Future research should investigate which measures best capture engagement. Another limitation of this study is that we only measured positive emotions after the study and not before. Lastly, this was an exploratory experiment with a relatively small sample size that did not achieve the number of participants deemed necessary based on the power analysis. Further, the participants might have had a different reason to use the intervention than the intended users of the intervention. Owing to these limitations, the results should be interpreted with caution.

### Conclusions

To conclude, this study suggests that gamification, in a single session, may have a positive direct impact on involvement, flow, and the emotions “interest” and “inspiration.” Thereby, the gamified intervention seemed to be able to increase cognitive engagement and the combination of cognitive and affective engagement but not behavioral and affective engagement alone. To conclude, we cannot say that gamification “works” but that the design of an intervention, in this case gamification, can have an impact on how participants experience the intervention. Although a gamified design has the potential to make Web-based mental health interventions more meaningful and relevant to its participants, it is possible that this design needs to be different for different people in different settings. Future research should investigate how to match the design of an intervention to the setting, motivation, and preferences of participants.

The fact that the design can increase cognitive engagement and impact the meaningfulness and relevance of an intervention may be especially beneficial within the context of Web-based (mental) health interventions; working on one’s own well-being is important but may not necessarily need to be fun. Cognitive engagement is an important part of engagement, which is seen as an important predictor of both adherence to Web-based interventions and the effectiveness of these interventions [14,16,51]. This study is a first step in uncovering how gamification, and design in general, may enhance engagement in the context of psychological Web-based interventions and offers a starting point for creating engaging interventions.

## Abbreviations

IMI: Intrinsic Motivation Inventory
PANAS: Positive and Negative Affect Schedule
PII: Personal Involvement Inventory
PPL-FSQ: Flow State Questionnaire of the Positive Psychology Lab
SUS: System Usability Scale

## References

[1] Johansson R, Andersson G (2012). Internet-based psychological treatments for depression. Expert Rev Neurother.

[2] Webb T, Joseph Judith, Yardley Lucy, Michie Susan (2010). Using the internet to promote health behavior change: a systematic review and meta-analysis of the impact of theoretical basis, use of behavior change techniques, and mode of delivery on efficacy. J Med Internet Res.

[3] Cuijpers P, van Straten Annemieke, Andersson Gerhard (2008). Internet-administered cognitive behavior therapy for health problems: a systematic review. J Behav Med.

[4] Andersson G, Cuijpers P (2008). Pros and cons of online cognitive-behavioural therapy. Br J Psychiatry.

[5] Griffiths F, Lindenmeyer A, Powell J, Lowe P, Thorogood M (2006). Why are health care interventions delivered over the internet? A systematic review of the published literature. J Med Internet Res.

[6] Arnberg F, Linton Steven J, Hultcrantz Monica, Heintz Emelie, Jonsson Ulf (2014). Internet-delivered psychological treatments for mood and anxiety disorders: a systematic review of their efficacy, safety, and cost-effectiveness. PLoS One.

[7] Baumeister H, Reichler L, Munzinger M, Lin J (2014). The impact of guidance on Internet-based mental health interventions: A systematic review. Internet Interventions.

[8] Bolier L, Haverman M, Kramer J, Westerhof GJ, Riper H, Walburg JA, Boon B, Bohlmeijer E (2013). An Internet-based intervention to promote mental fitness for mildly depressed adults: randomized controlled trial. J Med Internet Res.

[9] Kelders SM, Bohlmeijer ET, Pots WTM, van GJEWC (2015). Comparing human and automated support for depression: Fractional factorial randomized controlled trial. Behav Res Ther.

[10] Spijkerman M, Pots W T M, Bohlmeijer E T (2016). Effectiveness of online mindfulness-based interventions in improving mental health: A review and meta-analysis of randomised controlled trials. Clin Psychol Rev.

[11] Christensen H, Griffiths Kathleen M, Farrer Louise (2009). Adherence in internet interventions for anxiety and depression. J Med Internet Res.

[12] Kelders Saskia. M., Kok Robin. N., Ossebaard Hans C, Van Gemert-Pijnen Julia E W C (2012). Persuasive system design does matter: a systematic review of adherence to web-based interventions. J Med Internet Res.

[13] Donkin L, Christensen Helen, Naismith Sharon L, Neal Bruce, Hickie Ian B, Glozier Nick (2011). A systematic review of the impact of adherence on the effectiveness of e-therapies. J Med Internet Res.

[14] Donkin L, Glozier Nick (2012). Motivators and motivations to persist with online psychological interventions: a qualitative study of treatment completers. J Med Internet Res.

[15] Kelders S (2015). Involvement as a Working Mechanism for Persuasive Technology, in Persuasive Technology. Springer International Publishing.

[16] Perski O, Blandford Ann, West Robert, Michie Susan (2017). Conceptualising engagement with digital behaviour change interventions: a systematic review using principles from critical interpretive synthesis. Transl Behav Med.

[17] Ludden G, van Rompay Thomas J L, Kelders Saskia M, van Gemert-Pijnen Julia E W C (2015). How to Increase Reach and Adherence of Web-Based Interventions: A Design Research Viewpoint. J Med Internet Res.

[18] Hamari J., Koivisto J., Sarsa H. (2014). Does gamification work? - A literature review of empirical studies on gamification.

[19] Waite-Jones JM, Majeed-Ariss R, Smith J, Stones SR, Van RV, Swallow V (2018). Young People's, Parents', and Professionals' Views on Required Components of Mobile Apps to Support Self-Management of Juvenile Arthritis: Qualitative Study. JMIR Mhealth Uhealth.

[20] Pope L, Garnett B, Dibble M (2017). Engaging Adolescents to Inform the Development of a Mobile Gaming App to Incentivize Physical Activity. JMIR Res Protoc.

[21] Leinonen A, Pyky R, Ahola R, Kangas M, Siirtola P, Luoto T, Enwald H, Ikäheimo TM, Röning J, Keinänen-Kiukaanniemi S, Mäntysaari M, Korpelainen R, Jämsä T (2017). Feasibility of Gamified Mobile Service Aimed at Physical Activation in Young Men: Population-Based Randomized Controlled Study (MOPO). JMIR Mhealth Uhealth.

[22] Deterding S, Dixon D, Khaled R, Nacke L (2011). From game design elements to gamefulness: defining gamification.

[23] Ahtinen A, Mattila E, Välkkynen P, Kaipainen K, Vanhala T, Ermes M, Sairanen E, Myllymäki T, Lappalainen R (2013). Mobile mental wellness training for stress management: feasibility and design implications based on a one-month field study. JMIR Mhealth Uhealth.

[24] Thorsteinsen K, Vittersø Joar, Svendsen Gunnvald Bendix (2014). Increasing physical activity efficiently: an experimental pilot study of a website and mobile phone intervention. Int J Telemed Appl.

[25] Johnson D, Deterding S, Kuhn K, Staneva A, Stoyanov S, Hides L (2016). Gamification for health and wellbeing: A systematic review of the literature. Internet Interventions.

[26] Mekler E, Brühlmann F, Tuch An, Opwis K (2017). Towards understanding the effects of individual gamification elements on intrinsic motivation and performance. Computers in Human Behavior.

[27] Sailer M, Hense Ju, Mayr Sk, Mandl H (2017). How gamification motivates: An experimental study of the effects of specific game design elements on psychological need satisfaction. Computers in Human Behavior.

[28] Appleton J.J., Christenson S.L., Furlong M.J. (2008). Student engagement with school: Critical conceptual and methodological issues of the construct. Psychology in the Schools.

[29] Graffigna G (2017). Is a Transdisciplinary Theory of Engagement in Organized Settings Possible? A Concept Analysis of the Literature on Employee Engagement, Consumer Engagement and Patient Engagement. Front Psychol.

[30] Archambault I, Janosz M, Morizot J, Pagani L (2009). Adolescent behavioral, affective, and cognitive engagement in school: relationship to dropout. J Sch Health.

[31] Zaichkowsky J (1985). Measuring the involvement construct. Journal of Consumer Research.

[32] Ryan RM, Deci EL (2000). Self-determination theory and the facilitation of intrinsic motivation, social development, and well-being. Am Psychol.

[33] Rigby S., Ryan R.M. (2011). Glued to games: How video games draw us in and hold us spellbound.

[34] Fredrickson BL (2004). The broaden-and-build theory of positive emotions. Philos Trans R Soc Lond B Biol Sci.

[35] Csikszentmihalyi M (1997). Flow and the Psychology of Discovery and Invention.

[36] Jegers K (2007). Pervasive game flow: understanding player enjoyment in pervasive gaming. Computers in Entertainment (CIE).

[37] Keyes CLM, Dhingra SS, Simoes EJ (2010). Change in level of positive mental health as a predictor of future risk of mental illness. Am J Public Health.

[38] Lamers S, Westerhof Gj, Glas Ca, Bohlmeijer Et (2015). The bidirectional relation between positive mental health and psychopathology in a longitudinal representative panel study. The Journal of Positive Psychology.

[39] Duckworth Angela Lee, Steen Tracy A, Seligman Martin E P (2005). Positive psychology in clinical practice. Annu Rev Clin Psychol.

[40] Ryff C (2014). Psychological well-being revisited: advances in the science and practice of eudaimonia. Psychother Psychosom.

[41] Schotanus-Dijkstra M, Drossaert CHC, Pieterse ME, Boon B, Walburg JA, Bohlmeijer ET (2017). An early intervention to promote well-being and flourishing and reduce anxiety and depression: A randomized controlled trial. Internet Interventions.

[42] Ludden G.D.S., Kelders S.M., Snippert B.H.J., Spagnolli A., Chittaro L., Gamberini L. (2014). This is your life!. PERSUASIVE 2014. Lecture Notes in Computer Science, vol 8462.

[43] Bohlmeijer E, Hulsbergen M (2013). Dit is jouw leven. Ervaar de effecten van de positieve psychologie (This is Your Life. Experience the Effects of Positive Psychology).

[44] Watson D, Clark LA, Tellegen A (1988). Development and validation of brief measures of positive and negative affect: the PANAS scales. J Pers Soc Psychol.

[45] McAuley E, Duncan T, Tammen VV (1989). Psychometric properties of the Intrinsic Motivation Inventory in a competitive sport setting: a confirmatory factor analysis. Res Q Exerc Sport.

[46] Magyaródi T, Nagy H, Soltész P, Mózes T, Oláh A (2013). Psychometric properties of a newly established flow state questionnaire. The Journal of Happiness & Well-Being.

[47] Brooke J (1996). SUS-A quick and dirty usability scale. Usability evaluation in industry.

[48] Hayes A (2017). Introduction to mediation, moderation, and conditional process analysis: A regression-based approach. Guilford Publications.

[49] Boendermaker WJ, Prins PJM, Wiers RW (2015). Cognitive Bias Modification for adolescents with substance use problems--Can serious games help?. J Behav Ther Exp Psychiatry.

[50] Sieverink F, Kelders S M, van Gemert-Pijnen J E W C (2017). Clarifying the Concept of Adherence to eHealth Technology: Systematic Review on When Usage Becomes Adherence. J Med Internet Res.

[51] Ritterband LM, Thorndike FP, Cox DJ, Kovatchev BP, Gonder-Frederick LA (2009). A behavior change model for internet interventions. Ann Behav Med.

